# Expanding the Range of Possibility: Exploring the Uncertainty in Projecting Ozone-Related Health Effects of Climate Change

**DOI:** 10.1289/ehp.120-a436a

**Published:** 2012-11-01

**Authors:** Bob Weinhold

**Affiliations:** Bob Weinhold, MA, has covered environmental health issues for numerous outlets since 1996. He is a member of the Society of Environmental Journalists.

Shifts in concentrations of ground-level ozone (O_3_) are one of the projected outcomes of climate change, although estimates of the magnitudes, locations, and impacts of these shifts vary. A team of researchers assessed several prediction scenarios of climate change between 2000 and 2050 to see how different modeling choices affect estimates of O_3_-related human health effects [*EHP* 120(11):1559–1564; Post et al.]. The goal of this study was to explore the extent of the uncertainty that surrounds different modeling choices.

The team evaluated seven climate change models (and their embedded assumptions about meteorology and atmospheric chemistry), each linked to an air quality model. They also evaluated five population projections as well as concentration–response relationships (i.e., anticipated adverse health effects of different O_3_ levels) based on selected epidemiological studies used to support the U.S. Environmental Protection Agency’s National Ambient Air Quality Standards for O_3_. The climate change models defined the “O_3_ season” as June–August, although most epidemiological studies of O_3_-related health effects typically assume a longer season of May–September. This suggested to the researchers that their health effect estimates may be conservative.

Three-fourths of the 105 scenarios predicted an increase in nonaccidental O_3_-related summertime deaths in the lower 48 states, but in a wide range, from 10 to 2,560; 23% of the scenarios predicted a decrease in deaths ranging from 10 to 650, and 3% predicted no change. Roughly parallel findings—with wide ranges of effects, and most scenarios predicting worsening outcomes—were seen for estimated morbidity impacts such as lost school days and minor restricted-activity days. There were large differences in both magnitude and direction in predicted health effects at the regional scale.

The researchers attributed the greatest variations in predicted health effects to differences in the linked climate change–air quality models, although the population models and concentration–response relationships also had significant impacts on the final estimates.

Consistent with any predictive effort that involves significant uncertainty, the researchers conclude that researchers and policy analysts should similarly consider multiple prediction scenarios for each component of their analyses, enabling a better assessment of the range of possible effects. That approach contrasts with previous projections of climate change/O_3_-related health impacts cited by the researchers, all of which utilized much narrower ranges of inputs than the current study.

